# Back to normal? A retrospective study on stimulation test and endocrinological diagnosis before, during and after COVID-19 pandemics

**DOI:** 10.3389/fendo.2025.1571685

**Published:** 2025-05-05

**Authors:** Martina Peinkhofer, Sofia Passarella, Paolo Dalena, Gianluca Tamaro, Viviana Vidonis, Giada Vittori, Daniela Slama, Elena Faleschini, Egidio Barbi, Gianluca Tornese

**Affiliations:** ^1^ University of Trieste, Trieste, Italy; ^2^ Institute for Maternal and Child Health IRCCS “Burlo Garofolo”, Trieste, Italy

**Keywords:** precocious puberty, growth hormone deficiency, stimulation tests, COVID-19, central precocious puberty (CPP)

## Abstract

**Introduction:**

The COVID-19 pandemic disrupted healthcare systems, affecting consultations and diagnostics. In pediatric endocrinology, referral patterns shifted, with increased central precocious puberty (CPP) diagnoses and decreased growth hormone deficiency (GHD) evaluations. This study examines trends in stimulation tests, referrals, and diagnoses from 2019 to 2023 to assess the pandemic’s impact on pediatric endocrinology.

**Methods:**

This retrospective study analyzed stimulation tests performed at the Institute for Maternal and Child Health IRCCS “Burlo Garofolo,” Trieste, Italy, from 2019 to 2023, divided into pre-pandemic (2019–March 2020), pandemic (March 2020–January 2022), and post-pandemic (February 2022–December 2023) phases.

**Results:**

A total of 1,526 tests were conducted on 1,042 patients. Tests per day remained stable (pre-pandemic: 0.84; pandemic: 0.82; post-pandemic: 0.85). The Arginine Stimulation Test (ATT), the most frequent test pre-pandemic (31%), decreased during the pandemic (20%, p<0.001), while the LHRH Test (LHRHT) increased from 22% to 28% (p<0.001), becoming the most requested test. Diagnosis rates for GHD and CPP remained stable, but the proportion of females diagnosed with CPP increased significantly during the pandemic (91% vs. 69%, p=0.022). CPP testing declined (p=0.018) post-pandemic, while GHD testing returned to pre-pandemic levels.

**Conclusion:**

The pandemic altered diagnostic patterns, with reduced GHD evaluations reflecting limited healthcare access. Post-pandemic recovery suggests a resolution of diagnostic delays. The temporary surge in CPP cases, likely influenced by pandemic-related lifestyle changes, subsided post-pandemic, aligning with pre-pandemic trends. To date, no other studies have reported similar variations in GHD incidence during the pandemic.

## Introduction

The COVID-19 pandemic significantly impacted healthcare systems, leading to a substantial reduction in medical consultations, both in emergency rooms and outpatient clinics (1, 2). In pediatrics, this was largely due to parental fears of viral exposure (3, 4).

In pediatric endocrinology, approximately half of initial referrals are typically related to the evaluation of short stature or early puberty (5). During the pandemic, changes in referral patterns and diagnoses have been described. In particular, many centers have noticed an important increase in referrals and diagnoses of central precocious puberty (CPP) in girls since the early months of 2020 (6); since puberty onset is regulated by both genetic and environmental factors, several potential triggers—such as stress and sudden lifestyle changes (e.g., increased screen time, dietary variations, altered sleep habits)—have been hypothesized to contribute to this trend (7–12). Parents also may have observed pubertal signs more readily while spending more time at home. In contrast, referrals for short stature and diagnoses of growth hormone deficiency (GHD) decreased, likely due to postponed pediatric visits (13).

In the following years, the improvement of containment measures and the development of the anti-SARS-CoV-2 vaccination campaign enabled the gradual resumption of routine hospital activities (14), eventually returning to pre-pandemic levels. However, it remained unclear whether diagnostic patterns previously reported in pediatric endocrinology returned to pre-pandemic trends or persisted, potentially exhibiting a “dragging” effect.

This study aimed to assess the evolution of stimulation tests and endocrine diagnoses from 2019 to 2023, focusing on the persistence or reversal of pandemic-related changes, especially in CPP and GHD.

## Materials and methods

We conducted a retrospective study at the Institute for Maternal and Child Health IRCCS “Burlo Garofolo” in Trieste, Italy, a tertiary hospital and research institute that serves as a pediatric referral center for the province of Trieste and as a national reference hospital.

All records of children and adolescents who underwent a stimulation test from January 1, 2019, to December 31, 2023, were reviewed. To better understand how the pandemic impacted the frequency and distribution of tests and diagnoses, we categorized the analyzed five-year period (2019–2023) into three distinct time intervals:

- the *pre-pandemic period*: January 1, 2019, to March 7, 2020 (the day before the nationwide lockdown in Italy) (15);
- the *pandemic period*: March 8, 2020, to January 31, 2022, during which hospital access was restored (16), allowing the return to daily activities even for individuals without a “Green Pass”;
- the *post-pandemic period*: February 1, 2022, to December 31, 2023.

Since these three periods differ in duration, corrections were applied based on the number of days in each period to ensure a standardized comparison of test frequencies and diagnoses.

To access all patients’ data, we used the “G2 Clinico” platform (a management system for specialist activities). We collected information on age at presentation, sex, height Standard Deviation Score (SDS), BMI SDS, reasons for referral, type and number of tests performed, test results, and final diagnosis, as previously described (17).

Ethical Committee approval was not required, as the General Authorization to Process Personal Data for Scientific Research Purposes (Authorization no. 9/2014) states that retrospective archive studies using ID codes, which prevent data from being traced back directly to the subject, do not need ethics approval (18).

Parents provided informed consent at the first visit, agreeing that “clinical data may be used for clinical research purposes, epidemiology, the study of pathologies, and training, to improve knowledge, care, and prevention.”

The statistical analyses were primarily descriptive. Data were presented as absolute and percentage frequencies and were described using means or medians and interquartile ranges (IQRs), depending on their distribution. The two-proportions Z-test was used to evaluate associations between two proportions, while Mood’s median test was applied to assess differences in the medians of two groups for continuous variables. All tests were two-tailed, and a p-value <0.05 was considered statistically significant. Analyses were performed using R 4.4.2 (2024, R Core Team, Vienna, Austria).

## Results

Over the five-year study period, a total of 1,526 stimulation tests were performed on 1,042 individuals, 55% of whom were female. Among these, 270 individuals (26%) underwent more than one test. The year with the lowest number of tests was 2020, with 266 tests, while the highest numbers were recorded in 2021 and 2023, with 328 tests each. Overall, 474 tests (31%) yielded pathological results.

The most frequently performed test overall was the Arginine Tolerance Test (ATT), accounting for 367 tests (24%), followed by the Luteinizing Hormone-Releasing Hormone Test (LHRHT) (n = 346, 23%), the Standard Dose Synacthen Test (SDST) (n = 309, 20%), and the Insulin Tolerance Test (ITT) (n = 290, 19%).

The primary reasons for performing stimulation tests included short stature (370 tests, 24%, as the first stimulation test; 163 tests, 11%, as a second stimulation test), precocious pubarche (n = 309, 20%), and precocious puberty (n = 252, 16%).

### Trends in stimulation tests over time

Analyzing the three time periods described above, we observed a stable trend in the number of tests performed (**Table 1**):

- in the *pre-pandemic period*, a total of 361 tests were conducted over 432 days, corresponding to an average of 0.84 tests per day.
- during the *pandemic period*, 569 tests were performed across 695 days, resulting in an average of 0.82 tests per day.
- in the *post-pandemic period*, 596 tests were conducted over 699 days, with an average of 0.85 tests per day.

**Table 1:** Summary of stimulation tests conducted and individuals tested from 2019 to 2023.

Variable	Pre-pandemic period	Pandemic period	Post-pandemic period	Total
*Days (n)*	432	695	699	1826
*Tests (n)*	361	569	596	1526
*Tests per day (n/day)*	0.84	0.82	0.85	0.84
*Individuals (n)*	245	389	408	1042
*Female (n, %)*	195 (54%) ^B^	349 (61%) ^A C^	296 (50%) ^B^	840 (55%)
*More than 1 test performed (n,%)*	67 (27%)	108 (27%)	95 (23%)	270 (26%)

A: significantly different from pre-pandemic period; B: significantly different from pandemic period; C: significantly different from post-pandemic period.

Given the differences in duration among the three periods, corrections were applied based on the number of days in each period to ensure a standardized comparison of test frequencies and diagnoses.

Period-to-period comparisons of the average number of tests per day showed no significant differences, indicating that despite the restrictions, our service continued to provide consistent clinical care. The distribution of stimulation tests and pathological responses is reported in **Table 2**.

**Table 2:** Distribution of stimulation test conducted from 2019 to 2023 and rate of pathological response.

Test	Pre-pandemic period	Pandemic period	Post-pandemic period	Total
**ATT**	112 (31%) ^B C^	113 (20%) ^A^	142 (24%) ^A^	367 (20%)
*Female (n, %)*	47 (42%)	49 (43%)	50 (35%)	146 (40%)
*Tests per day (n/day)*	0.26	0.16	0.20	
*Pathological tests (n, %)*	46 (41%)	56 (50%)	66 (46%)	168 (46%)
**LHRH**	79 (22%) ^B^	160 (28%) ^A C^	107 (18%) ^B^	346 (19%)
*Female (n, %)*	59 (75%)	128 (80%)	78 (73%)	265 (77%)
*Tests per day (n/day)*	0.18	0.23	0.15	
*Pathological tests (n, %)*	34 (43%)	59 (37%)	33 (31%)	126 (36%)
**SDST**	64 (18%)	128 (22%)	117 (20%)	309 (17%)
*Female (n, %)*	49 (77%)	105 (82%)	91 (78%)	245 (79%)
*Pathological tests (n, %)*	0 (0%)	2 (2%)	2 (1%)	4 (1%)
**ITT**	33 (9%)	49 (9%)	63 (11%)	145 (8%)
*Female (n, %)*	13 (40%)	18 (37%)	24 (38%)	55 (38%)
*Pathological tests (n, %)*	27 (82%)	39 (80%)	55 (87%)	121 (83%)
**ATT+GHRHT (R)**	19 (5%)	35 (6%)	37 (6%)	91 (5%)
*Female (n, %)*	6 (32%)	18 (51%)	9 (24%)	33 (36%)
*Pathological tests (n, %)*	0 (0%)	3 (9%)	4 (11%)	8 (9%)
**LDST**	20 (6%)	22 (4%)	19 (3%)	61 (3%)
*Female (n, %)*	8 (40%)	8 (36%)	4 (21%)	20 (33%)
*Pathological tests (n, %)*	1 (5%)	2 (9%)	3 (16%)	6 (10%)
**ITT (RETESTING)**	0	0	18 (3%)	18 (1%)
*Female (n, %)*			7 (39%)	7 (39%)
*Pathological tests (n, %)*			8 (44%)	8 (44%)
**ATT+GHRHT**	1 (<1%)	4 (1%)	10 (2%)	15 (1%)
*Female (n, %)*	0 (0%)	1 (25%)	2 (20%)	3 (20%)
*Pathological tests (n, %)*	1 (100%)	2 (50%)	3 (30%)	6 (40%)
**CTT**	0	2 (<1%)	4 (1%)	6 (<1%)
*Female (n, %)*		1 (50%)	2 (50%)	3 (50%)
*Pathological tests (n, %)*		2 (100%)	2 (50%)	4 (67%)
**TRHT**	0	4 (1%)	0	4 (<1%)
*Female (n, %)*		1 (25%)		1 (25%)
*Pathological tests (n, %)*		2 (50%)		2 (50%)
**CRHT**	0	2 (<1%)	0	2 (<1%)
*Female (n, %)*		2 (100%)		2 (100%)
*Pathological tests (n, %)*		0 (0%)		0 (0%)

A: significantly different from pre-pandemic period; B: significantly different from pandemic period; C: significantly different from post-pandemic period.

### Changes in test distribution

- In the *pre-pandemic period*, the most frequently conducted test was the ATT (n = 112, 31%), followed by the LHRHT (n = 79, 22%) and the SDST (n = 64, 18%).
- During the *pandemic period*, we observed a shift in this trend: the most commonly performed test was the LHRHT (n = 160, 28%), followed by the SDST (n = 128, 22%) and the ATT (n = 113, 20%).
- In the *post-pandemic period*, the ATT returned as the most frequently conducted test (n = 142, 24%), followed by the SDST (n = 117, 20%) and the LHRHT (n = 107, 18%).

The proportion of ATT tests relative to the total number of tests significantly decreased during the pandemic compared to the pre-pandemic period (from 31% to 20%, p < 0.001) and remained significantly lower in the post-pandemic period compared to pre-pandemic levels (31% to 24%, p = 0.018).

**Figure 1:**
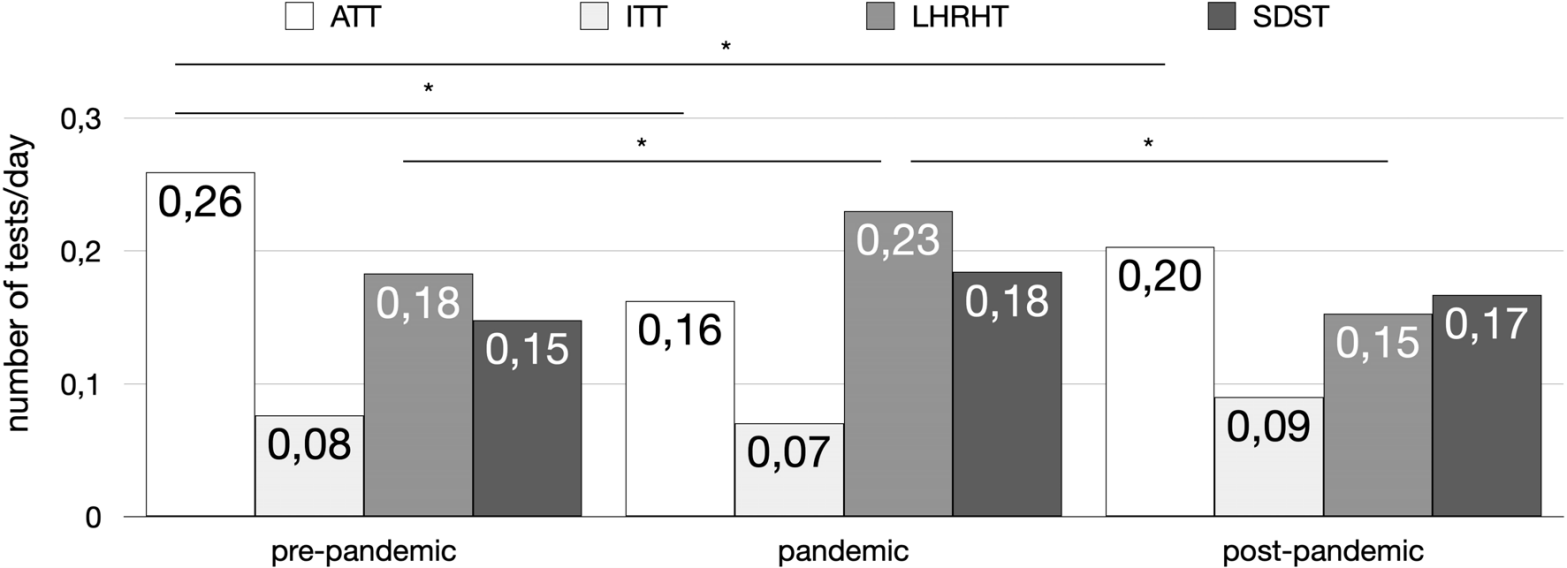
Distribution of the principal stimulation tests (number of tests per day) across the three time periods (pre-pandemic, pandemic, and post-pandemic). The tests included: Arginine Tolerance Test (ATT) (white bars), Insulin Tolerance Test (ITT) (light gray bars), Luteinizing Hormone-Releasing Hormone Test (LHRHT) (medium gray bars), and Standard Dose Synacthen Test (SDST) (dark gray bars). Asterisks (*) indicate statistically significant differences (p < 0.05) between groups.

Conversely, the proportion of LHRHT tests increased significantly during the pandemic compared to the pre-pandemic period (22% to 28%, p = 0.041) and remained significantly higher in the pandemic period compared to the post-pandemic period (18%, p < 0.001), while there was no significant difference between the pre-pandemic and post-pandemic periods (22% to 18%, p = 0.160) (**Figure 1**).

No significant differences were observed for the remaining tests, likely due to their lower frequency of execution.

### Sex distribution of tested patients

Females were more frequently tested than males in all three time periods. Additionally, a significant increase in the proportion of females undergoing testing was observed during the pandemic period, compared to both the pre-pandemic period (61.3% vs. 54%, p = 0.027) and the post-pandemic period (61.3% vs. 49.7%, p < 0.001).

However, the proportion of females tested in the pre- and post-pandemic periods was similar (49.7% vs. 54%, p = 0.205). This increase was likely attributable to the higher number of LHRHT tests performed in females during the pandemic period.

### Evaluation and diagnosis of growth hormone deficiency

- During the *pre-pandemic period*, 146 tests for suspected GHD were conducted on 107 individuals, with an average of 0.25 individuals tested per day. Tests for suspected GHD accounted for 40% of the total tests performed.
- In the *pandemic period*, the number of tests and individuals tested per day significantly decreased, with 168 tests performed on 113 individuals over 695 days, corresponding to an average of 0.16 individuals tested per day. These tests represented 30% of the total tests performed.
- In the *post-pandemic period*, 219 tests were conducted on 163 individuals, representing 37% of the total tests performed, with an average of 0.23 individuals tested per day.

**Figure 2:**
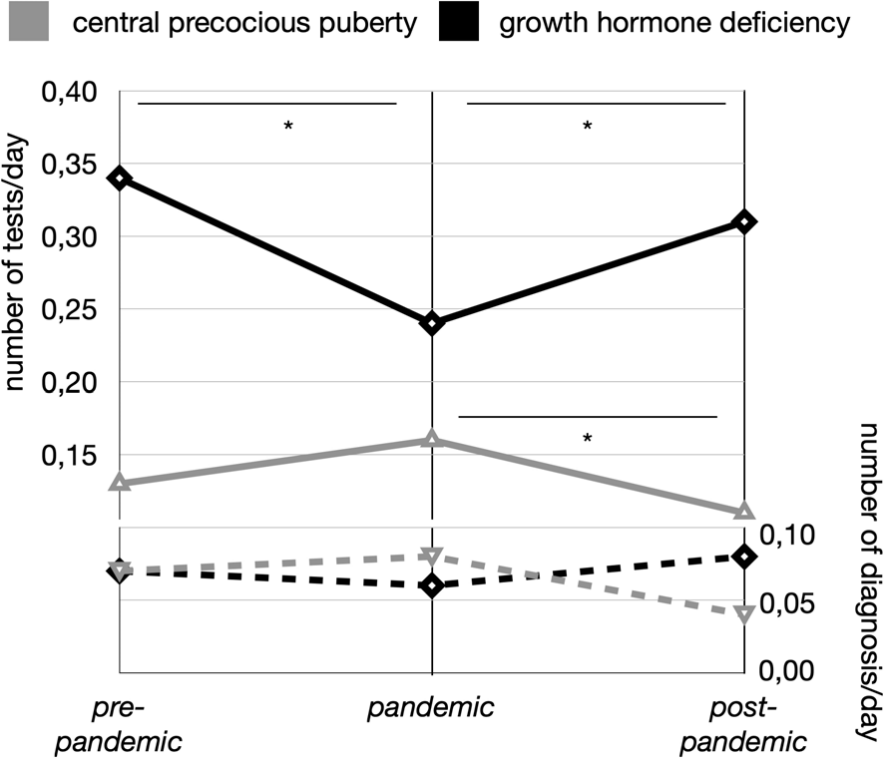
Trends in stimulation tests and diagnoses for central precocious puberty (CPP) and growth hormone deficiency (GHD) across the three study periods (pre-pandemic, pandemic, and post-pandemic). Solid lines represent the number of stimulation tests per day, with gray indicating CPP and black indicating GHD. Dashed lines represent the number of diagnoses per day, with gray for CPP and black for GHD. Asterisks (*) indicate statistically significant differences (p < 0.05) between time periods.

A significant difference in the proportion of individuals tested for suspected GHD was observed between the pre-pandemic and pandemic periods (p < 0.001) and between the post-pandemic and pandemic periods (p = 0.002) (**Figure 2**). In contrast, the number of individuals tested for suspected GHD was similar between the pre-pandemic and post-pandemic periods (p = 0.394), indicating that significantly fewer individuals were tested for suspected GHD during the pandemic period.

Similar results were observed when considering the number of tests for suspected GHD performed per day (p < 0.001 between pre-pandemic and pandemic periods, p = 0.003 between pandemic and post-pandemic periods, p = 0.426 between pre-pandemic and post-pandemic periods).

Despite these differences, no significant variations were observed in the proportion of GHD diagnoses across the three periods:

- 32 individuals were diagnosed with GHD *pre-pandemic* (30%),
- 42 during the *pandemic* (37%), and
- 57 *post-pandemic* (35%).

Likewise, the number of diagnoses per day did not differ significantly across the three periods (0.07/day pre-pandemic, 0.06/day pandemic, 0.08/day post-pandemic) (**Figure 2**).

No significant differences were found in sex distribution or age at presentation among the tested children across the three periods. Similarly, there were no significant differences in the median height of tested children, although the post-pandemic period had the lowest median height at diagnosis (-2.15 SDS).

Details regarding GHD tests are reported in **Table 3**.

**Table 3:** Distribution of individuals tested for growth hormone deficiency (GHD).

Variable	Pre-pandemic period	Pandemic period	Post-pandemic period
**Test conducted for suspect GHD** (n, %)	146 (40%)	168 (30%)	219 (37%)
*- Test per day (n/day)*	0.34 ^B^	0.24 ^A C^	0.31 ^B^
**Individuals tested for suspect GHD** (n, %)	107 ^B^	113 ^A C^	163 ^B^
*- Individuals tested per day (n/day)*	0.25	0.16	0.23
*- Female (n, %)*	45 (42%)	50 (44%)	57 (35%)
*- Age (years) (median, IQR)*	12.1 (9.2;13.3)	11.82 (9.2;13.4)	12.25 (10.1;13.7)
*- Height (SDS) (median, IQR)*	-2.17 (-2.57;-1.72)	-2.09 (-2.66;-1.55)	-2.18 (-2.56;-1.69)
**GHD diagnosis** (n, %)	32 (30%)	42 (37%)	57 (35%)
*- Diagnosis per day (n/day)*	0.07	0.06	0.08
*- Female (n, %)*	12 (37%)	16 (38%)	23 (40%)
*- Age at diagnosis (years) (median, IQR)*	12.3 (10.6-;13.5)	12.0 (10.0;13.5)	12.1 (10.1;13.8)
*- Height at diagnosis (SDS) (median, IQR)*	-1.98 (-2.50 -1.46)	-1.93 (-2.53; -1.51)	-2.15 (-2.56; -1.74)

A: significantly different from pre-pandemic period; B: significantly different from pandemic period; C: significantly different from post-pandemic period.

### Evaluation and diagnosis of central precocious puberty

Among individuals tested for suspected CPP, the highest proportion was observed during the pandemic period. Specifically:

- in the *pre-pandemic period*, 57 tests for suspected CPP were performed, accounting for 16% of the total tests, with an average of 0.13 tests per day.
- during the *pandemic period*, the number of tests increased to 113, representing 20% of the total tests, with an average of 0.16 tests per day.
- in the *post-pandemic period*, the number of tests decreased to 82, accounting for 14% of the total tests, with an average of 0.11 tests per day.

A significant difference was observed in the proportion of individuals tested for suspected CPP between the pandemic and post-pandemic periods (p = 0.0043), indicating that significantly more individuals were tested for suspected CPP during the pandemic compared to the post-pandemic period (**Figure 2**).

Regarding individuals diagnosed with CPP:

- 29 diagnoses were made *pre-pandemic* (51%).
- 55 diagnoses were made during the *pandemic* (49%).
- 31 diagnoses were made *post-pandemic* (47%).

No significant differences were observed in the proportion of CPP diagnoses across the three periods, nor in the number of diagnoses per day (**Figure 2**). Further details regarding CPP testing are provided in **Table 4**.

**Table 4:** Distribution of individuals tested for central precocious puberty (CPP).

Variable	Pre-pandemic period	Pandemic period	Post-pandemic period
**Test conducted for suspect CPP** (n, %)	57 (16%)	113 (20%)	82 (14%)
*- Individual tested per day (n/day)*	0.13	0.16 ^C^	0.11 ^B^
*- Female (n, %)*	46 (81%)	92 (81%)	65 (79%)
*- Age (years) (median, IQR)*	7.85 (7.42;8.85)	7.98 (7.47;8.84)	7.81 (7.26;8.48)
*- Height (SDS) (median, IQR)*	1.03 (0.44-1.79	1.04 (0.40-1.75)	1.01 (0.51;1.72)
*- BMI (SDS) (median, IQR)*	0.44 (-0.17-0.97)	0.32 (-0.31-0.97)	0.10 (-0.69-0.96)
**CPP diagnosis** (n, %)	29 (51%)	55 (49%)	31 (37%)
*- Diagnosis per day (n/day)*	0.07	0.08	0.04
*- Female (n, %)*	20 (69%) ^B^	50 (91%) ^A^	24 (77%)
*- Age at diagnosis (years) (median, IQR)*	8.85 (8.01;9.55) ^B C^	8.15 (7.62;8.97) ^A^	7.98 (7.40-8.87) ^A^
*- Height at diagnosis (SDS) (median, IQR)*	1.45 (0.79;2.00)	0.87 (0.23;1.79)	0.9 (0.51;1.61)
*- BMI at diagnosis (SDS) (median, IQR)*	0.44 (0.11-0.97) ^B C^	0.22 (-0.53-0.82) ^A^	0.23 (-0.55;1.06) ^A^
*- Bone age at diagnosis (SDS) (median, IQR)*	11.00 (10.00-11.66)	10.00 (8.83-11.00)	10.00 (8.43-11.00)

A: significantly different from pre-pandemic period; B: significantly different from pandemic period; C: significantly different from post-pandemic period.

No significant differences were found among tested patients in terms of age at presentation or median height at presentation. However, BMI at presentation in patients tested for CPP was significantly higher in the pre-pandemic period compared to both the pandemic and post-pandemic periods.

Among patients diagnosed with CPP, an overall decrease in the median age at diagnosis was observed:

- a significant difference was found between the pre-pandemic period (8.85 years; 8.01–9.55) and the pandemic period (8.15 years; 7.62–8.97) (p = 0.022).
- a further significant difference was observed between the pre-pandemic period and the post-pandemic period, where the lowest median age at diagnosis was recorded (7.98 years; 7.40–8.87) (p = 0.010).
- in contrast, the median age at diagnosis was similar between the pandemic and post-pandemic periods.

The highest median bone age at diagnosis of CPP was reported during the pre-pandemic period (11.00 years; 10.00–11.66), which was significantly higher than during the pandemic period (10.00 years; 8.83–11.00) (p = 0.012). However, no significant difference in median bone age at diagnosis was found between the pandemic and post-pandemic periods (10.00 years; 8.43–11.00).

Throughout the entire study period, females were tested for CPP more frequently than males. A total of 252 tests for CPP were conducted, of which 203 were in females (80.5%). The total number of CPP diagnoses during the study period was 115, with 94 of these cases occurring in females (82%).

- *Pre-pandemic period:* 46 females were tested (81% of total CPP tests), and 20 diagnoses were made (69% of CPP diagnoses).
- *Pandemic period:* 92 females were tested (81%), and 50 received the diagnosis (91% of CPP diagnoses).
- *Post-pandemic period:* 65 females were tested (79%), and 24 diagnoses were made (77% of CPP diagnoses).

While the proportion of females tested remained consistent across the three periods, the prevalence of females diagnosed with CPP was highest during the pandemic period (91%), which was significantly higher than in the pre-pandemic period (69%, p = 0.0239). No significant difference was found in the prevalence of females diagnosed between the pandemic and post-pandemic periods, although the prevalence decreased post-pandemic (from 91% to 77%, p = 0.158). Likewise, no significant difference was observed between the pre-pandemic and post-pandemic periods (from 69% to 77%, p = 0.654).

## Discussion

In this retrospective study, we analyzed endocrinological stimulation tests conducted at a tertiary pediatric center over a five-year period (2019–2023), focusing on the impact of the COVID-19 pandemic on diagnostic patterns. Our findings provide novel insights into how healthcare disruptions affected diagnostic activities and how these patterns evolved in the post-pandemic period.

Across the full study period, the number of tests performed per day remained stable, indicating that despite pandemic-related restrictions, our service continued to provide uninterrupted care. However, the pandemic led to notable shifts in referral patterns. Previously published data from our center had shown a significant reduction in testing for GHD during the pandemic and an increase in CPP-related referrals, particularly among females (13). Our expanded dataset confirms these trends and shows a clear return to pre-pandemic diagnostic patterns (“back to normal”) in the subsequent years.

### GHD: a silent diagnostic drop

We observed a significant decline in the number of GHD evaluations during the COVID-19 pandemic, followed by a return to pre-pandemic levels. Specifically, our data show that the daily number of individuals tested for GHD was comparable before (0.25/day) and after the pandemic (0.23/day), but significantly lower during the pandemic period (0.16/day, *p* < 0.005). This reduction—also reported in our previous study, where a 35% drop in GHD-related tests and a 30% reduction in confirmed diagnoses in 2020 compared to 2019 were observed (13)—is likely attributable to the suspension of routine well-child visits, parental reluctance to seek non-urgent medical care, and reduced opportunities for caregivers to monitor and compare children’s growth trajectories with their peers. The widespread implementation of remote schooling and the near-total suspension of extracurricular activities further limited social interactions, potentially delaying the recognition of growth delays.

Although this temporary decline raised concerns about missed opportunities for timely diagnosis, the daily number of confirmed GHD diagnoses remained stable across the three periods (0.07/day pre-pandemic, 0.06/day during the pandemic, and 0.08/day post-pandemic). In the present analysis, the pandemic period spanned from March 8, 2020, to January 31, 2022, (from the nationwide lockdown in Italy to the full restoration of free access to daily activity, including hospital access). Compared to our previous study, which focused primarily on the peak pandemic phase of 2020, the extended observation window likely captured a partial recovery of diagnostic activities. Indeed, although the number of evaluations remained lower, the percentage of confirmed diagnoses among those tested was higher during the pandemic (37%) and post-pandemic periods (35%) compared to the pre-pandemic period (30%), albeit without reaching statistical significance. This suggests that despite reduced screening, children referred during the pandemic may have presented with more overt clinical signs, leading to a higher diagnostic yield and possibly mitigating the risk of missed diagnoses.

While the impact of the pandemic on CPP diagnosis has been widely investigated, its effect on GHD evaluation remains underexplored. Our findings therefore provide a novel contribution to addressing this gap in the current literature. Notably, disparities in GHD evaluation and treatment are deeply influenced by structural and socio-demographic factors, including caregiver perceptions and provider biases. The pandemic may have exacerbated these disparities by disproportionately limiting healthcare access and delaying referrals—especially among populations already underserved. Our data reinforce the need to ensure equitable and timely access to growth monitoring and endocrine evaluations, particularly in times of healthcare system disruption​ (19).

### CPP: a confirmed international spike

In our study, we observed a clear increase in CPP-related diagnostic activity during the COVID-19 pandemic, both in terms of testing frequency and confirmed diagnoses. Specifically, the proportion of stimulation tests performed for suspected CPP increased from 14–16% in the pre- and post-pandemic periods to 20% during the pandemic. The number of daily CPP tests also rose significantly during the pandemic (from 0.13/day pre-pandemic to 0.16/day), with a predominant increase among girls.

This trend is supported by a robust body of international literature documenting similar increases in CPP diagnoses across diverse populations and healthcare settings. Reports from Italy (6, 13, 20–28), Germany​ (29), Portugal (30), Spain (31) Turkey (32–35), Lebanon (36), Japan (37), South Korea (38)​, Thailand (39), Singapore (40), China​ (41, 42), India (43), the United States​ (44–46), Argentina (47, 48) and Brazil (49) consistently describe this phenomenon. In some of these studies, the number of CPP diagnoses more than doubled compared to previous years—for example, a 5.01-fold increase reported by Fu et al. (42) and a 2.3-fold increase by Geniuk et al. (48)—underscoring the global nature of this shift.

Interestingly, in our cohort, the proportion of confirmed CPP diagnoses among those tested remained stable across all periods (0.07/day pre-pandemic, 0.08/day during the pandemic, 0.04/day post-pandemic), suggesting that the rise in diagnoses was not attributable to overtesting or relaxed diagnostic thresholds. Rather, the data point toward a genuine increase in incidence—particularly among females. Indeed, the proportion of females diagnosed with CPP rose significantly during the pandemic (from 69% pre-pandemic to 91%, *p* = 0.0043), before decreasing to 77% in the post-pandemic period. Notably, this increase was observed exclusively among females, with no significant change in diagnoses among males, aligning with previous reports (48, 50). This finding reinforces the idea that male CPP may be less influenced by environmental factors and is more often associated with organic or genetic causes (51, 52).

Unlike our study, which showed stable BMI trends across the observed periods, several reports noted increased BMI among children diagnosed with CPP during the pandemic (6, 23, 36, 39, 40). However, other studies (21, 43, 46, 48) found no significant differences in BMI, suggesting that lifestyle factors—beyond weight gain alone—may be at play.

Importantly, our data extend beyond the acute phase of the pandemic, capturing a return to pre-pandemic referral and diagnosis patterns by 2022–2023. To date, only two published studies have included longitudinal post-pandemic data, making our study one of the few to explore trends in the normalization of CPP care. Vargas Trujillo et al. reported a doubling of CPP cases requiring puberty suppression during the pandemic, followed by a gradual decline in the post-pandemic period (46). Similarly, Chioma et al. observed an increase in progressive CPP diagnoses in 2020, which decreased in 2021 and returned to pre-pandemic levels by 2022 (22). Notably, Chioma et al. also documented a significant increase in screen time and a reduction in physical activity in 2020 compared to both 2019 and 2022. As pandemic restrictions eased, these environmental stressors diminished, and the surge in CPP cases subsided. These findings support the hypothesis that environmental factors—such as reduced physical activity, increased screen exposure, sleep disruption, and psychosocial stress—may have acted as triggers for earlier pubertal onset, particularly among girls (11). This also reinforces the notion that female puberty may be more sensitive to environmental influences.

Contrasting with the findings of Vargas Trujillo et al., who reported an increasing trend in both median age at diagnosis (from 7.1 years pre-pandemic, to 7.35 during the pandemic, and 8.01 post-pandemic) and bone age (from 9.5 to 10.4 years) (46), our data reveal a progressive *decrease* in median age at CPP diagnosis across the three periods (from 8.85 years pre-pandemic, to 8.15 during the pandemic, and 7.98 post-pandemic). One possible explanation for this discrepancy may lie in differences in referral timing: while Vargas Trujillo et al. interpreted the older age at diagnosis as the result of delayed access to care, our center may have succeeded in maintaining more timely evaluations despite external constraints. Alternatively, our findings might reflect broader secular trends toward earlier pubertal onset, which have been observed globally since the 1990s (53, 54). Notably, Chioma et al. did not report significant changes in age at diagnosis across study years, further underscoring the variability of pandemic-related effects across populations and healthcare systems.

This study has several strengths. First, it presents a comprehensive five-year analysis from a single tertiary pediatric center, covering the pre-pandemic, pandemic, and post-pandemic phases. By standardizing test frequency according to observation time, we minimized potential biases due to differences in period length. Additionally, by including both GHD and CPP, we provide a broader and more nuanced picture of how different endocrine conditions were impacted by healthcare disruptions and subsequently recovered.

While the increase in CPP diagnoses during the pandemic has been widely documented, few studies have explored the normalization phase in the post-pandemic period, and even fewer have assessed the temporary decline and subsequent recovery of GHD diagnostics. Our findings therefore contribute uniquely to both areas. From a clinical perspective, these results underscore the resilience and adaptability of pediatric endocrinology services, while also highlighting the need for targeted strategies to ensure timely referrals during healthcare crises.

Nonetheless, some limitations must be acknowledged. The retrospective design and single-center scope may limit the generalizability of our findings. Although we adjusted for differences in observation time, unmeasured confounding factors—such as seasonal variation in referrals or shifts in local healthcare policies—may have influenced the observed trends. Furthermore, while we hypothesize that environmental and behavioral changes contributed to the increased CPP referrals, we did not collect direct data on lifestyle factors such as screen time, dietary habits, physical activity, or psychosocial stress. This limits the strength of causal inferences. Prospective studies incorporating behavioral, metabolic, and hormonal assessments are needed to better elucidate these associations.

Finally, although our time-stratified analysis helps to distinguish pandemic-related trends from longer-term changes, it remains possible that broader secular shifts—both genetic and environmental—may also influence pubertal timing and growth. Future multicenter studies involving diverse populations, larger sample sizes, and long-term outcome data will be essential to validate and expand on our findings.

## Conclusion

The findings of this study highlight the significant, albeit temporary, impact of the COVID-19 pandemic on pediatric endocrinology diagnostic patterns, particularly for GHD and CPP. During the pandemic period, notable deviations in testing and diagnosis trends were observed. The post-pandemic recovery demonstrates the resilience and adaptability of healthcare systems in restoring routine activities. Although the critical phase of the pandemic is behind us, it remains highly relevant to reflect on how such a global event significantly altered daily habits and access to healthcare; examining its impact on diagnostic trends continues to offer valuable insights into system resilience and evolving patient priorities. The observed shifts in diagnostic patterns underscore the potential influence of environmental and lifestyle factors on endocrine conditions, warranting further investigation. These insights not only deepen our understanding of the pandemic’s repercussions on pediatric health but also emphasize the importance of maintaining accessible and proactive healthcare services during times of crisis to mitigate diagnostic delays and ensure timely interventions.

## Data Availability

The raw data supporting the conclusions of this article will be made available by the authors, without undue reservation.
